# Genome-Wide Identification of the *LcGA2ox* Gene Family in Litchi (*Litchi chinensis* Sonn.) and Its Functional Analysis in Gibberellin Metabolism and Reproductive Development

**DOI:** 10.3390/plants15060914

**Published:** 2026-03-16

**Authors:** Weinan Song, Fuchu Hu, Zhe Chen, Tingting Yan, Yukun He, Hongna Zhang, Boxing Shang

**Affiliations:** 1 Hainan Provincial Key Laboratory of Quality Control of Tropical Horticultural Crops, Sanya Institute of Breeding and Multiplication, School of Tropical Agriculture and Forestry, Hainan University, Haikou 570228, China; 18503483257@163.com; 2 Institute of Tropical Fruit Trees, Hainan Academy of Agricultural Sciences/Key Laboratory of Genetic Resources Evaluation and Utilization of Tropical Fruits and Vegetables (Co-Construction by Ministry and Province), Ministry of Agriculture and Rural Affairs/Key Laboratory of Tropical Fruit Tree Biology of Hainan Province, Haikou 571100, China

**Keywords:** *Litchi chinensis*, gibberellin, flower development, *LcGA2ox* gene family, bioinformatics analysis, expression pattern

## Abstract

Gibberellin 2-oxidase (GA2ox) is instrumental in gibberellin (GA) catabolism and the modulation of plant growth. In this investigation, nine *LcGA2ox* genes (*LcGA2ox1*-*LcGA2ox9*) were identified within the litchi (*Litchi chinensis* Sonn.) genome. Bioinformatics analyses revealed that the proteins encoded by these genes uniformly possess 2OG-Fe (II)_Oxy and DIOX_N domains, exhibiting a range of physicochemical properties and subcellular localizations. Phylogenetic analysis categorized these genes into three subgroups, C_19_-GA2ox-I, C_19_-GA2ox-II, and C_20_-GA2ox-I, with each subgroup characterized by specific motif compositions and gene structures. These gene promoters harbor *cis*-regulatory elements implicated in light signaling, hormonal pathways, abiotic stress responses, and developmental processes. The *LcGA2ox* gene family contributes to the maintenance of GA metabolic homeostasis through interactions with GA synthases, receptors, and repressors. This gene family demonstrates distinct tissue-specific and spatiotemporal expression patterns: *LcGA2ox1/6/7* are predominantly expressed in flowers, *LcGA2ox8* in fruits, and *LcGA2ox9* in buds. Notably, *LcGA2ox6/7* are key regulators of male and female flower development in litchi, exhibiting a negative correlation with the female flower genes *AGL1* and *SPT*. The overexpression studies conducted in *Arabidopsis* have demonstrated that *LcGA2ox6* acts as an inhibitor of both vegetative and reproductive development. This study characterizes the *LcGA2ox* family, establishing a theoretical basis for understanding GA regulation in litchi reproductive development and genetic improvement.

## 1. Introduction

Gibberellins (GAs) are crucial for plant flowering and the development of floral organs [1]. The homeostasis of GAs within plants is co-regulated by a variety of enzymes, with GA oxidases facilitating both their biosynthesis and catabolism. Among these, Gibberellin 2-oxidase (GA2ox), a key enzyme in active GA catabolism, collaborates with the biosynthetic rate-limiting enzymes GA20ox and GA3ox to modulate GA homeostasis in plants [2]. The inactivation of GAs is critically important for effectively modulating the concentration of active GAs and maintaining their balance within plants. During GA biosynthesis, in addition to the production of active GA1 and GA4, a series of intermediate products are also formed, including GA9, GA12, GA15, GA19, GA20, GA24, GA44, and GA53 [3]. GAs can be categorized based on the number of carbon atoms they contain into C_20_-GAs, including GA12, GA15, GA19, GA24, GA44, and GA53, and C_19_-GAs, such as GA1, GA4, GA9, and GA20 [4]. Several metabolic inactivation pathways exist for GAs, with 2β-hydroxylation mediated by GA2ox being the most significant and representative.

GA2ox proteins are classified according to their substrate preference into C_20_-GA2ox, which acts on C_20_-GAs, and C_19_-GA2ox, which targets C_19_-GAs [5]. Phylogenetic analysis further subdivides C_19_-GA2ox into two distinct subgroups [6,7,8], indicating potential functional divergence among subgroup members. When C_19_-GAs serve as substrates, GA2ox catalyzes the conversion of bioactive GA4 and GA1 into their inactive metabolites, GA34 and GA8, respectively. Additionally, the precursors GA9 and GA20 are transformed into the inactive forms GA51 and GA29. In contrast, when C_20_-GAs serve as substrates, GA2ox primarily targets upstream intermediates in the GA biosynthesis pathway, such as GA12, GA15, GA19, GA24, GA44, and GA53, converting them into inactive hydroxylated products like GA97 and GA110 through 2β-hydroxylation. This process effectively inhibits the synthesis of active C_19_-GAs at the source, thereby significantly reducing the overall GA levels in plants [9].

To date, *GA2ox* genes have been characterized in multiple plant species due to their critical involvement in development. In *Arabidopsis thaliana*, the regulation of root growth is achieved through the modulation of gibberellin (GA) levels in the elongation zone by *GA2ox6* and *GA2ox8* [6]. In rice (*Oryza sativa* L.), the class III *GA2ox* gene *OsGA2ox6* regulates plant height by utilizing C_20_-GAs as substrates, and its overexpression results in a dominant dwarf phenotype without impacting flowering and seed development, thus offering a valuable genetic resource for rice dwarf breeding [10]. Similarly, overexpression of the apple (*Malus domestica* Borkh.) *MdGA2ox8* gene significantly reduces internode length and induces dwarfing [11]. In *Prunus mume*, *PmGA2ox8* is predominantly expressed in stems, responds to indole-3-acetic acid (IAA) and GA3 signals, and its overexpression markedly inhibits internode elongation, resulting in a dwarf phenotype [12]. Furthermore, overexpression of peach (*Prunus persica* (L.) Batsch.) genes *PpGA2ox1*, *PpGA2ox5*, and *PpGA2ox2* in tobacco (*Nicotiana tabacum* L.) leads to dwarfism, stem shortening, and leaf size reduction. It is hypothesized that these mechanisms could be employed to control tree height and crown width in peach, thereby reducing pruning costs [13]. The overexpression of the tobacco *GAox* gene in *Kalanchoe blossfeldiana* has been shown to significantly reduce plant height by approximately 50% and enhance leaf chlorophyll content, thereby facilitating the breeding of compact ornamental plants [14].

The *GA2ox* gene also plays crucial regulatory roles during reproductive growth. In tomatoes (*Solanum lycopersicum* L.), the *SlGA2ox1* and *SlGA2ox2* genes influence fruit development and parthenocarpy by modulating ovary growth [15]. In grapes (*Vitis vinifera* L.), the transcript levels of *GA2ox* genes peak during the flowering period, with most members showing decreased expression post-fruit setting, except for *VvGA2ox4*, which is significantly upregulated. This upregulation is negatively correlated with active GA content, indicating its involvement in maintaining GA homeostasis during fruit setting [16]. In apples, *MdGA2ox8* exhibits the highest expression levels in flowers, and any functional abnormalities in this gene can result in delayed flowering [11]. Similarly, in *Prunus mume*, *PmGA2oxs* genes influence flowering time by regulating GA levels during flower bud dormancy [12]. Furthermore, in *Jatropha curcas*, the expression of *JcGA2ox6* driven by a flower-specific promoter results in a compact inflorescence structure, smaller floral organs, a reduced number of male and female flowers, and a decreased female flower ratio [17].

Within the genus Litchi, *Litchi chinensis* Sonn. is a major tropical and subtropical fruit tree, valued for its evergreen nature and economic importance [18]. The primary floral types of litchi are categorized into three developmental stages and two sexual morphologies: female flowers, characterized by well-developed ovaries and functional stigmas capable of normal pollination, exhibit slow stamen development and fulfill female reproductive roles; male flowers possess fully abortive pistils (ovaries) with functional stamens that perform male reproductive functions; and bisexual flowers, which initially develop ovaries that subsequently abort, maintain functional stamens for male reproductive roles [19]. In litchi cultivation, challenges such as inconsistent flowering and an imbalanced male-to-female flower ratio adversely impact yield enhancement and the sustainable growth of the industry [20]. Research indicates that the application of uniconazole reduces the overall flower count and the number of male flowers, while increasing the number of female flowers and improving the male-to-female flower ratio, thereby significantly enhancing both fruit quantity and fruit-setting rate per inflorescence [21]. The application of uniconazole during the panicle development stage of litchi results in a reduction of endogenous GA levels within the panicle, suggesting that the influence of uniconazole on flowering is intricately linked to the modulation of endogenous hormone levels and their ratios [22]. The research findings underscore the pivotal role of GA in regulating the differentiation of male and female floral structures in litchi plants. Nonetheless, the extent to which GA-related genes affect the flowering process and reproductive organ development in this species remains inadequately investigated.

This research successfully detected nine potential *LcGA2ox* genes in the litchi genome. A series of detailed bioinformatics investigations were performed, including examinations of molecular characteristics, genomic organization, cross-species synteny, regulatory elements, predicted protein interactions, transcriptional networks, and expression profiles of these genes, aiming to pinpoint those potentially involved in floral and fruit maturation processes. The results offer significant groundwork for subsequent exploration of gibberellin-mediated regulatory pathways in litchi reproductive development, while also contributing valuable insights for enhancing breeding strategies and agricultural practices related to litchi flowering and fruiting.

## 2. Results

### 2.1. Identification and Characterization of the LcGA2ox Gene Family in Litchi

This study employed a comprehensive approach to identify *LcGA2ox* family members. Using GA2ox proteins from *Arabidopsis* and rice as queries, a Blastp search was conducted against the litchi genome. Subsequent analysis of candidate sequences with NCBI-CDD and SMART databases confirmed the presence of the conserved 2OG-Fe(II)_Oxy domain, leading to the identification of nine *GA2ox* genes. These were designated *LcGA2ox1* through *LcGA2ox9* based on chromosomal location, and their encoded proteins’ physicochemical properties are detailed in Table 1. The LcGA2ox proteins exhibit an amino acid length ranging from 332 to 371 residues, with an average length of 347. Their predicted molecular weights fall between 37.4 and 41.0 kDa, and the theoretical isoelectric points (pI) span a range from 6.07 to 9.00. Subcellular localization predictions indicated that the potential localization sites of *LcGA2ox* family members are predominantly in the nucleus, cytoplasm, and chloroplast. Among the *LcGA2ox* gene family, LcGA2ox2 and LcGA2ox3 are predicted to be predominantly localized within the nucleus, whereas LcGA2ox1, LcGA2ox5, LcGA2ox8, and LcGA2ox9 are primarily distributed in the cytoplasm. In contrast, LcGA2ox4, LcGA2ox6, and LcGA2ox7 are anticipated to be mainly localized within the chloroplast. These localization predictions were derived from analyses conducted using the WoLF PSORT tool, which provides a foundational reference for subsequent experimental validation.

**Figure 1:**
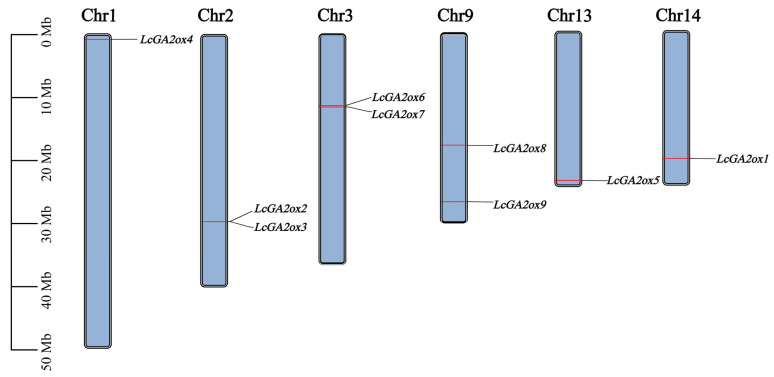
Chromosomal localization of *LcGA2ox* genes in litchi. *LcGA2oxs* are localized on chromosomes 1, 2, 3, 9, 13, and 14, and the chromosome length is in megabases (Mb).

Furthermore, chromosomal localization analysis utilizing litchi genome data revealed that the nine *GA2ox* genes in litchi are distributed across six chromosomes: chromosome 1 (Chr1), chromosome 2 (Chr2), chromosome 3 (Chr3), chromosome 9 (Chr9), chromosome 13 (Chr13), and chromosome 14 (Chr14) (Figure 1).

### 2.2. Classification of LcGA2oxs: C_19_-GA2ox-I, C_19_-GA2ox-II, and C_20_-GA2ox-I Subgroups

**Figure 2:**
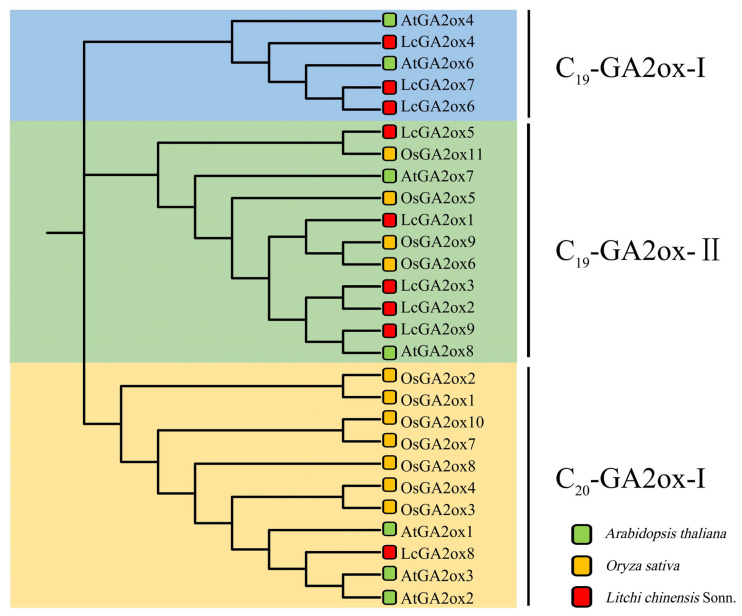
Phylogenetic tree inferred from predicted GA2ox amino acid sequences across species using MEGA5.1. Sequence alignment resulted in the delineation of three major subgroups (C_19_-GA2ox-I, C_19_-GA2ox-II, and C_20_-GA2ox-I), each represented by a unique color to facilitate clade identification.

GA2ox enzymes typically exhibit activity on either C_19_-GAs or C_20_-GAs, leading to their classification into C_19_-GA2ox and C_20_-GA2ox categories [5]. The presence of the three GA2ox subgroups (C_19_-GA2ox-I, C_19_-GA2ox-II, and C_20_-GA2ox-I) is a highly conserved characteristic among both monocotyledonous and dicotyledonous plant species [13]. In order to infer the functional roles of LcGA2ox enzymes, this study utilized identified GA2ox proteins from *Arabidopsis* and rice to construct a phylogenetic tree of the *LcGA2ox* family (Figure 2). The analysis revealed that the *GA2ox* family members are categorized into three distinct groups: C_19_-GA2ox-I, C_19_-GA2ox-II, and C_20_-GA2ox-I. Members of the C_19_-GA2ox-I and C_19_-GA2ox-II subgroups have been reported to utilize C_19_-GAs as substrates, whereas members of the C_20_-GA2ox-I subgroup predominantly act on C_20_-GAs. Specifically, *LcGA2ox4/6/7* are classified within the C_19_-GA2ox-I subgroup; *LcGA2ox1/2/3/5/9* are categorized under the C_19_-GA2ox-II subgroup; and *LcGA2ox8* is associated with the C_20_-GA2ox-I subgroup. This phylogenetic clustering suggests that *LcGA2ox1/2/3/4/5/6/7/9* are likely to exhibit activity on C_19_-GAs, while LcGA2ox8 is predicted to be active on C_20_-GAs.

### 2.3. Analysis of Gene Structure, Conserved Domains, and Conserved Motifs of the LcGA2ox Gene Family in Litchi

A comprehensive characterization of the structural attributes and conservation patterns of the *LcGA2ox* gene family in litchi was undertaken. Gene structure, conserved domains, and motifs were systematically examined using Tbtools-II v2.210 [23] to elucidate the commonalities and distinctions among LcGA2ox proteins.

**Figure 3:**
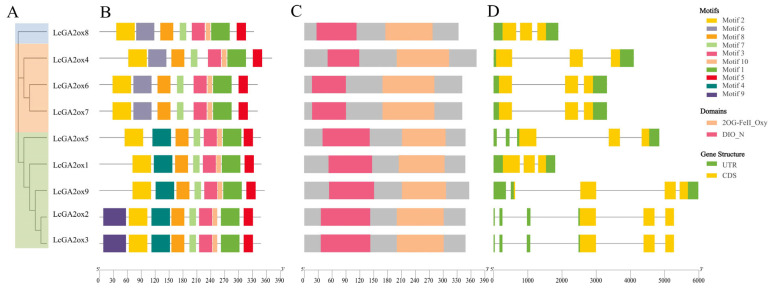
Characterization analysis of litchi *GA2ox* genes. (**A**) Phylogenetic analysis of LcGA2ox proteins constructed using the neighbour-joining method, illustrating evolutionary relationships and classification into distinct subgroups. (**B**) Distribution and organization of conserved motifs among the nine GA2ox proteins. Ten distinct colored boxes denote different motifs; motif positions were identified using MEME or related tools. (**C**) Domain architecture of the nine LcGA2ox proteins. All members contain the 2OG-Fe(II)_Oxy and DIOX_N domains, characteristic of GA2ox enzymes. (**D**) Gene structure analysis showing exon-intron organization of LcGA2ox genes. Yellow boxes represent coding sequences (CDS), green boxes indicate untranslated regions (UTRs), and connecting lines depict introns (non-coding segments).

To investigate the conservation and diversity of litchi *GA2ox* genes, potential motifs were predicted using the MEME Suite (https://meme-suite.org/meme/tools/meme, accessed on 12 September 2025), based on the phylogenetic relationships of the full-length sequences. The analysis identified ten motifs across all litchi GA2ox proteins, designated as motifs 1 through 10. Notably, Motif 9 was found exclusively in LcGA2ox2/3, Motif 6 was present only in LcGA2ox4/6/7/8, and Motif 4 was identified solely in LcGA2ox1/2/3/5/9. These findings suggest that Motif 4 is closely associated with the C_19_-GA2ox-II subgroup (Figure 3B).

A domain refers to a segment of a protein characterized by a specific function and the capacity to operate or move independently from other protein regions. Characterization of conserved domains demonstrated that all nine members of the litchi *GA2ox* family harbor both the 2OG-Fe(II)_Oxy and DIOX_N domains. Motifs 1, 3, 7, and 10 were found to be associated with the 2OG-Fe(II)_Oxy domain, within which the conserved motifs display high conservation across all family members. In contrast, variations exist in the conserved motifs of the DIOX_N domain among different family members. Specifically, the DIOX_N domain in LcGA2ox4/6/7/8 comprises Motifs 2 and 6; in LcGA2ox1/5/9, it consists of Motifs 2 and 4; and in LcGA2ox2/3, it includes Motifs 2, 4, and 9. These differences in motif composition may be directly linked to the functional specificity of the DIOX_N domain in substrate recognition (Figure 3C). Gene structure analysis indicates that, with the exception of LcGA2ox5/9, which contain four introns, and LcGA2ox2/3, which contain five introns, all other members have only two introns, highlighting the structural diversity within this family throughout evolution (Figure 3D).

### 2.4. Genomic Distribution and Evolutionary Relationships of LcGA2ox Genes in Litchi

**Figure 4:**
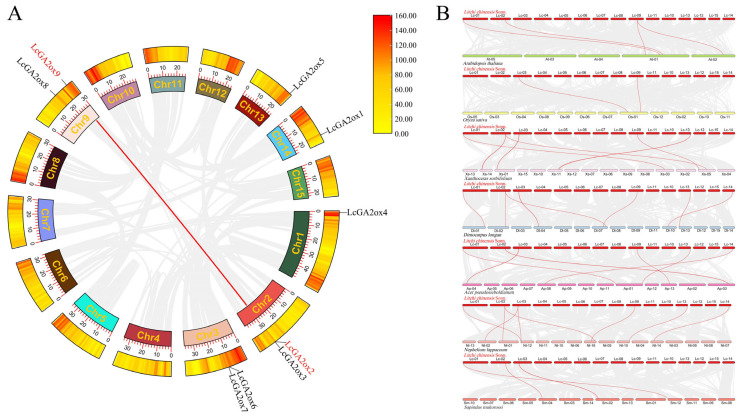
Collinearity analysis of litchi *GA2ox* genes. (**A**) Intragenomic collinearity of *LcGA2ox* genes within the litchi genome. Eight litchi chromosomes are arranged in a circular layout; grey lines indicate syntenic gene pairs across the genome, while red lines specifically highlight collinear relationships among *LcGA2ox* genes. (**B**) Inter-genomic collinearity and chromosomal distribution of *GA2ox* genes in litchi, *Arabidopsis*, rice, *Dimocarpus longan*, *Nephelium lappaceum*, *Sapindus mukorossi* Gaertn., *Xanthoceras sorbifolium*, and *Acer pseudosieboldianum*. Numbers represent chromosome numbers.

A collinearity analysis was conducted using Tbtools-II to investigate the evolutionary mechanisms underlying the *LcGA2ox* gene family in litchi. The results revealed a tandem duplication event between *LcGA2ox2* and *LcGA2ox9*, indicating that this duplication event may have played a role in the expansion of the *GA2ox* gene family (Figure 4A).

Furthermore, a comparative collinearity analysis was performed across litchi, *Arabidopsis*, rice, and five other Sapindaceae species: *Dimocarpus longan*, *Nephelium lappaceum*, *Sapindus mukorossi* Gaertn., *Xanthoceras sorbifolium*, and *Acer pseudosieboldianum*. The findings indicated that *LcGA2ox2* and *LcGA2ox4* exhibit collinearity with *GA2ox* genes in all six species, excluding rice; *LcGA2ox6* is collinear with *GA2ox* in the five Sapindaceae species; *LcGA2ox7* shows collinearity with *GA2ox* in all species except *Arabidopsis*; and *LcGA2ox9* is collinear with *GA2ox* in all species except *Sapindus mukorossi* Gaertn. These results suggest that these *LcGA2ox* genes have an ancient evolutionary origin and may have retained relatively conserved functions throughout evolutionary history (Figure 4B).

### 2.5. Analysis of Cis-Acting Elements in the Promoter Regions of LcGA2ox Genes in Litchi

**Figure 5:**
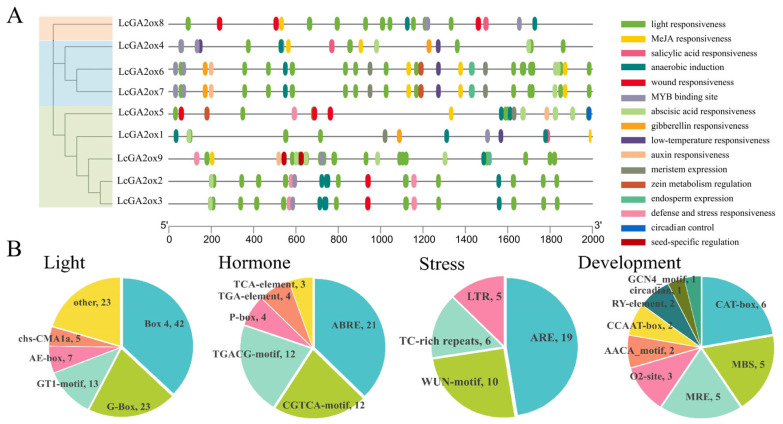
*Cis*-acting elements analysis in *GA2ox* promoters. (**A**) Classification and distribution of predicted *cis*-acting elements within the promoter regions of *LcGA2ox* genes. (**B**) Pie chart illustrating the proportional abundance of different *cis*-acting elements categorized into four major response types: phytohormone-responsive, stress-responsive, light-responsive, and development-related elements.

In order to explore the possible roles of *GA2ox* genes in litchi plants, researchers isolated a 2000 base pair segment located upstream of the ATG initiation codon, which was designated as the promoter region. This DNA fragment was then subjected to computational analysis through the PlantCARE web-based tool (available at http://bioinformatics.psb.ugent.be/webtools/plantcare/html/, accessed on 14 September 2025). The examination identified multiple *cis*-regulatory elements distributed throughout these promoter sequences (Figure 5).

The analysis revealed 16 distinct *cis*-acting regulatory elements linked to various biological processes, including light response, hormone signaling, stress adaptation, and developmental control. Within the light-responsive category, Box 4 elements showed the highest frequency, being detected 42 times. For hormonal regulation, the ABRE element responsive to abscisic acid emerged as the most abundant, found in 21 copies. The study also identified 12 copies each of two MeJA-responsive motifs (CGTCA and TGACG). Among stress-related elements, the ARE sequence involved in anaerobic response was most prominent, occurring 19 times. In developmental regulation elements, the CAT-box motif related to meristem activity appeared most frequently, with 6 copies identified.

The analysis identified significant diversity in the types and distribution of *cis*-acting elements within the *LcGA2ox* gene promoters. Notably, all *GA2ox* genes, with the exception of *LcGA2ox3*, possess MeJA response elements; all *GA2ox* genes, except for *LcGA2ox4*, contain ABA response elements; and all *GA2ox* genes, excluding *LcGA2ox2/5*, include GA response elements.

The findings suggest that *GA2ox* genes in litchi may be critically involved in regulating a range of physiological processes, including photoperception, multiple phytohormone signaling cascades, environmental adaptation mechanisms, and morphogenetic processes.

### 2.6. Prediction Analysis of Protein–Protein Interactions of LcGA2ox in Litchi

**Figure 6:**
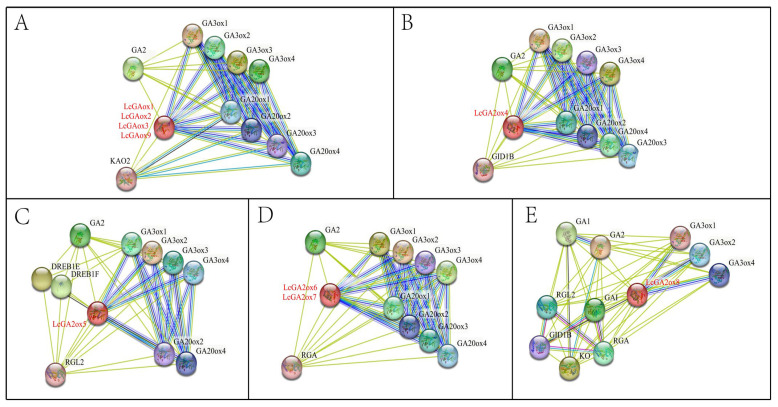
Predicted regulatory network of LcGA2ox and its interacting proteins. (**A**) Predicted regulatory network of LcGA2ox1/2/3/9 and its interacting proteins. (**B**) Predicted regulatory network of LcGA2ox4 and its interacting proteins. (**C**) Predicted regulatory network of LcGA2ox5 and its interacting proteins. (**D**) Predicted regulatory network of LcGA2ox6/7 and its interacting proteins. (**E**) Predicted regulatory network of LcGA2ox8 and its interacting proteins. The network integrates multiple lines of evidence: sky blue lines indicate database-derived interactions; purple lines represent experimentally validated associations; dark blue lines denote gene co-occurrence; green lines reflect text-mining results; black lines indicate co-expression; and light blue lines signify protein homology.

In the subsequent analysis, the prediction of protein–protein interactions of LcGA2ox in litchi was conducted using *Arabidopsis* as a reference for homologous protein sequences. The STRING database was leveraged to predict gene homology and reconstruct protein–protein interaction (PPI) networks, thereby shedding light on the potential functional roles of *LcGA2ox* genes (Figure 6). The results indicated that all members are predicted to interact with the GA synthase GA3ox family, while all members, except LcGA2ox8, are predicted to interact with the GA synthase GA20ox family. Furthermore, all members are predicted to interact with inactive GA2, with only LcGA2ox8 predicted to interact with both active GA1 and inactive GA2.

The protein LcGA2ox4 is predicted to interact with the GA signaling receptor GID1B [24]. LcGA2ox5 is anticipated to form a potential interaction network with the GA signaling repressor RGL2 and stress-responsive factors DREB1E/F [25]. Proteins LcGA2ox1/2/3/9 are predicted to interact with KAO2, a core enzyme involved in GA biosynthesis [26]. Additionally, LcGA2ox6/7 are expected to establish a potential interaction network with the GA signaling repressor RGA [27]. LcGA2ox8 is predicted to interact with GA signaling repressors RGL2, GAI, and RGA, as well as the GA receptor GID1B and KO, a crucial enzyme regulating the initial steps of the GA biosynthetic pathway [24,27,28,29]. It should be emphasized that these interactions are predicted bioinformatically and require subsequent experimental validation.

The metabolic balance of GAs is achieved through the coordinated regulation of synthases and the inactivating enzyme GA2ox. This interaction network elucidates the central role of the *GA2ox* family in litchi GA metabolism and signaling pathways, providing a crucial reference for further analysis of the molecular mechanisms by which *GA2ox* regulates physiological processes, including litchi GA levels.

### 2.7. Expression Pattern Analysis of LcGA2ox Genes in Litchi

#### 2.7.1. Comparative Analysis of *LcGA2ox* Gene Expression Across Litchi Tissues

**Figure 7:**
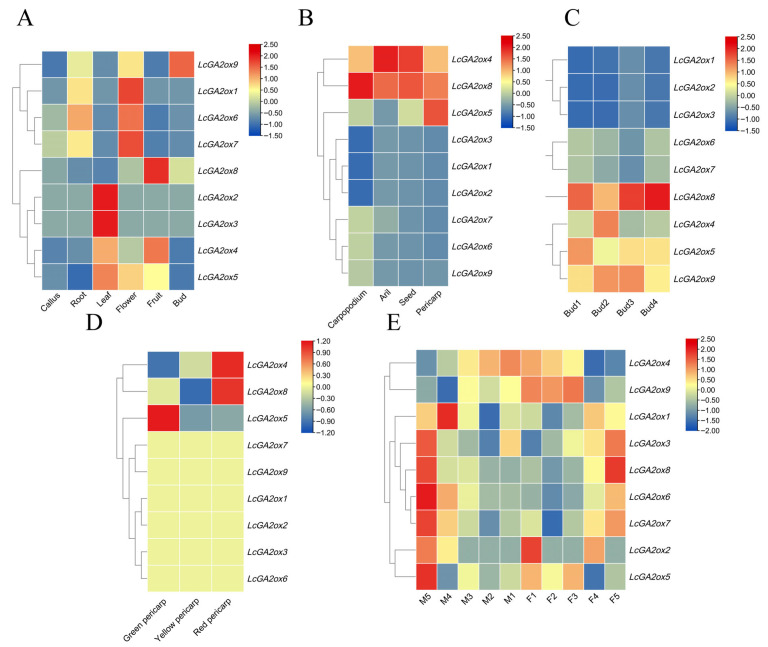
Transcriptome-based expression profiling of *LcGA2ox* genes in ‘Feizixiao’ litchi. (**A**) Organ specific expression of *LcGA2ox* genes in ‘Feizixiao’ Litchi. (**B**) Spatial expression dynamics in various fruit tissues. (**C**) Temporal expression during leaf bud development. (**D**) Expression trajectories across pericarp coloring stages. (**E**) Differential expression in male and female flower development stages (F1–F5: female flower stages; M1–M5: male flower stages). All samples were subjected to three independent biological replicates, and data were normalized prior to heatmap generation.

To explore the potential roles of the *LcGA2ox* gene family, transcriptome profiling was conducted using RNA-seq data derived from the ‘Feizixiao’ litchi cultivar. The results revealed distinct tissue-specific expression patterns among family members across six different organs, including callus, root, leaf, inflorescence, fruit, and bud (Figure 7A and Table S1). Specifically, *LcGA2ox2* and *LcGA2ox3* were predominantly expressed in leaves, *LcGA2ox1*, *LcGA2ox6*, and *LcGA2ox7* were notably expressed in flowers, *LcGA2ox8* was highly expressed in fruits, and *LcGA2ox9* was predominantly expressed in buds. The results imply functional diversification among *LcGA2ox* members, with specific roles in the growth and development of particular litchi tissues.

#### 2.7.2. Differential Expression of *LcGA2ox* Family Members Across Litchi Fruit Organs

Further analysis of expression patterns in pedicel, aril, seed, and pericarp was conducted using transcriptome data. The results demonstrated significant differential expression of this gene family in various fruit tissues (Figure 7B and Table S2). *LcGA2ox4* and *LcGA2ox8* were highly expressed in pedicel, aril, and seed, while *LcGA2ox4*, *LcGA2ox5*, and *LcGA2ox8* were highly expressed in pericarp. Other family members did not exhibit significant expression across all fruit tissues. These findings suggest distinct roles for *LcGA2ox* members: *LcGA2ox4* and *LcGA2ox8* in gibberellin-mediated fruit development, and *LcGA2ox5* in pericarp-specific processes.

#### 2.7.3. Expression Patterns of *LcGA2oxs* in Four Developmental Stages of Litchi Leaf Buds

The expression patterns across four developmental stages of ‘Feizixiao’ litchi leaf buds were examined utilizing transcriptome data. Significant differential expression was observed for certain gene family members during the four stages of leaf bud development (Figure 7C and Table S3). Notably, *LcGA2ox8* demonstrated significant expression throughout all four stages, with its expression levels in the first, third, and fourth stages markedly surpassing those of other family members. By contrast, significant expression of *LcGA2ox4* was detected solely during the second stage, whereas *LcGA2ox5* was highly expressed only at the initial stage, and *LcGA2ox9* demonstrated notable expression in the subsequent two stages. These observations suggest that *LcGA2ox8* is implicated in the regulation of GA metabolism throughout the entirety of leaf bud development, playing a crucial role in morphogenesis and growth differentiation. Meanwhile, *LcGA2ox4*, *LcGA2ox5*, and *LcGA2ox9* appear to function at distinct critical periods of leaf bud development, collectively facilitating the orderly progression of leaf bud development.

#### 2.7.4. Expression Patterns of *LcGA2oxs* During Pericarp Coloring of Litchi

The expression patterns during the pericarp coloring process in ‘Feizixiao’ litchi were examined through transcriptomic analysis. The findings revealed that various members of this gene family exhibited significant differential expression across the three stages of pericarp coloring (Figure 7D and Table S4). In particular, *LcGA2ox4* and *LcGA2ox8* exhibited elevated transcriptional activity in the fruit outer layer during maturation to red pigmentation, whereas *LcGA2ox5* demonstrated notable expression exclusively in unripe green fruits. A striking observation was the gradual upregulation of *LcGA2ox4* transcript levels in the pericarp throughout the developmental sequence from green to yellow to red coloration phases. These findings imply that *LcGA2ox4* could participate in biochemical mechanisms related to fruit skin maturation and pigment accumulation through sustained regulation of GA metabolic pathways. The data further indicate that *LcGA2ox8* likely serves important functions in advanced ripening phases, while the early-stage expression profile of *LcGA2ox5* points to its possible involvement in controlling GA concentrations during initial fruit development.

#### 2.7.5. Expression Patterns of *LcGA2oxs* During the Development of Male and Female Flowers of Litchi

Transcriptomic analysis of ‘Feizixiao’ litchi floral development (Figure 7E and Table S5) demonstrated distinct temporal expression profiles for *LcGA2ox* gene family members. Notably, *LcGA2ox4* showed prominent transcriptional activity specifically in the bisexual flower bud phase, prior to complete sexual differentiation. The investigation identified *LcGA2ox5* and *LcGA2ox9* as being predominantly active in initial phases of pistillate flower formation (F1 and F2 stages), whereas *LcGA2ox1* displayed marked expression restricted to early staminate flower development (M1 and M2 stages). Later developmental phases exhibited differential expression patterns, with *LcGA2ox6* and *LcGA2ox7* becoming transcriptionally active during advanced staminate flower maturation (M3 and M4 stages), while *LcGA2ox8* expression peaked exclusively in terminal pistillate flower development (F4 stage). These results indicate that *LcGA2ox4* plays a pivotal role in the initiation of sexual differentiation mechanisms. Furthermore, *LcGA2ox5/9* appears to play a crucial role in the early developmental stages of female flowers, specifically in pistil primordium development and carpel differentiation, by precisely modulating GA levels at the onset of female flower development. In addition, *LcGA2ox8* may facilitate the maturation of female floral characteristics, such as pistil shaping and stigma cracking, by adjusting GA levels during the later stages of female flower development. In male flowers, *LcGA2ox1* is implicated in early developmental processes, including stamen primordium differentiation and initial filament development, through the regulation of GA metabolism in the early stages of male flower development. Collectively, our findings suggest that *LcGA2ox6* and *LcGA2ox7* are likely involved in the maturation of male flowers, particularly in anther maturation and pollen formation, through the regulation of GA metabolism during the late developmental stages of male flowers in *Litchi chinensis*.

#### 2.7.6. Expression Patterns of *LcGA2oxs* in Different Floral Organs of Litchi

**Figure 8:**
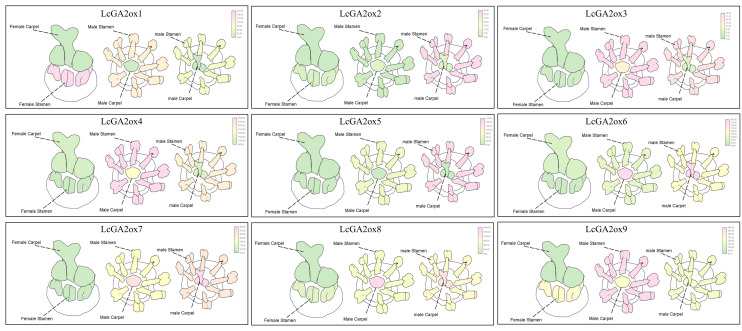
Expression patterns of *LcGA2ox* genes in different male and female floral organs of litchi. Red modules represent highly expressed tissues, green modules represent lowly expressed tissues, and all heatmaps are visualized using TBtools-II. All RNA-seq samples were run in three independent biological replicates, and the data were standardised before use for heatmap construction.

Transcriptome data were utilized to investigate the expression profiles of *LcGA2ox* genes within the floral organs of ‘Feizixiao’ litchi (Figure 8 and Table S6). The analysis indicated that *LcGA2ox5* was significantly expressed solely in the stamens of both male and bisexual flowers. Conversely, *LcGA2ox6/7* were prominently expressed in both the stamens and carpels of male and bisexual flowers. It is posited that *LcGA2ox5* plays a pivotal role in maintaining a “low GA environment” necessary for stamen development by locally degrading active GAs within the stamens. This degradation ensures critical functions such as anther maturation and pollen formation, thereby facilitating proper stamen development, which includes enhanced pollen viability and filament elongation, while preventing the transformation of stamens into pistils. Additionally, *LcGA2ox6/7* are hypothesized to create distinct GA environments and energy allocation patterns between male and female flowers, as evidenced by their high expression in male and bisexual flowers and low expression in female flowers. This differential expression likely influences the direction of floral sex differentiation in litchi. It is noteworthy that all *LcGA2ox* genes exhibit low expression levels in the pistils of female flowers (Female Carpel), potentially due to the pistils’ requirement for elevated GA levels to sustain their prolonged growth and functional development during subsequent reproductive processes.

### 2.8. Co-Expression Analysis of Key LcGA2ox Genes

To investigate the regulatory mechanisms of floral differentiation in litchi plants through GA metabolic pathway regulation during the bisexual floral bud phase, we performed comprehensive gene expression profiling. WGCNA was performed to systematically characterize gene expression dynamics during male and female flower development. The study focused on the most variably expressed 8000 genes, which were selected based on their median absolute deviation (MAD) scores for module construction. Through detailed examination of module-trait relationships with floral developmental stages, critical *LcGA2ox* genes involved in this process were successfully identified. The parameters and criteria employed in the WGCNA were as follows: the soft-thresholding power was set to 5 to calculate soft connectivity; an unsigned network was constructed; the minimum module size was defined as 100; the module merging threshold was determined using a module cuttree height of 0.25. Pearson correlation was utilized for correlation analysis, and co-expression weights between genes were assigned based on the adjacency matrix values calculated by Cytoscape. Bonferroni correction was applied for multiple test correction.

**Figure 9:**
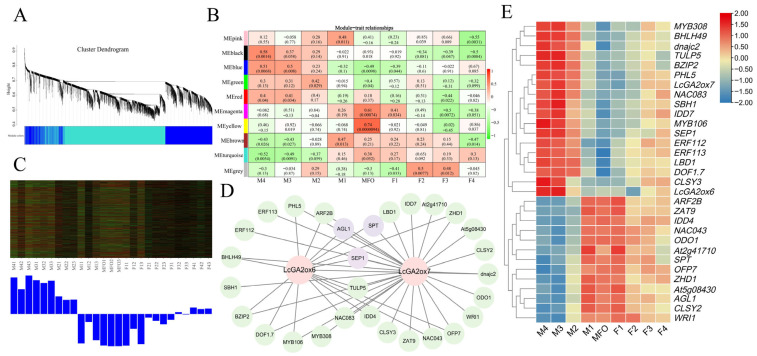
Co-expression network analysis of litchi flower development. (**A**) WGCNA module clustering dendrogram. (**B**) Trait-module eigengene correlation. (**C**) Module eigengene expression profiles. (**D**) Co-expression network of *LcGA2ox6/7* and TFs in the blue module. (**E**) Expression dynamics of *LcGA2ox* genes and TFs (MF0: early undifferentiated stage; F1–F5: female flower stages; M1–M5: male flower stages). Data are from three biological replicates and were z-score normalized for heatmap visualization.

In this study, eight co-expression modules were identified (Figure 9A), each comprising genes with positive and negative correlations. Notably, the blue module demonstrated a significant positive correlation with multiple stages of male flower development, specifically stages M3 and M4 (M3: r = 0.5, *p* = 0.008 < 0.01; M4: r = 0.51, *p* = 0.0068 < 0.01) (Figure 9B). The r-values represent the Pearson correlation coefficients between module eigengenes and traits, while the *p*-values have been adjusted for multiple testing of module-trait associations. The observed association suggests that *LcGA2ox6/7*, members of the *LcGA2ox* gene family identified in this module, could potentially influence the developmental progression of male litchi flowers (Figure 9C). WGCNA module annotation via GO and KEGG demonstrated a significant enrichment of genes in two central biological pathways: the transmission of plant hormone signals and the production of diterpenoid compounds, which are fundamental building blocks for GA formation (Figure S1). These findings lead to the proposition that the identified gene cluster might contribute to sexual differentiation in litchi flowers by participating in hormonal signaling mechanisms and regulating the production of GAs. Subsequent analysis identified that *LcGA2ox6/7* exhibit a strong co-expression relationship with 29 transcription factors (weight > 0.35) (Figure 9D and Tables S7 and S8). The term “weight” refers to the adjacency matrix value between genes, as calculated by Cytoscape. Among these transcription factors, 16, including *SEP1* (*LITCHI019333*), are highly positively correlated with *LcGA2ox6/7* and are significantly overexpressed in male flowers. Conversely, 13 transcription factors, such as *AGL1* (*LITCHI026995*) and *SPT* (*LITCHI010952*), are highly negatively correlated with *LcGA2ox6/7* and are significantly overexpressed in female flowers (Figure 9E and Table S9).

### 2.9. Functional Analysis of LcGA2ox6 Genes in Litchi

To further elucidate the functional role of *LcGA2ox6* in the regulation of plant growth and development, PCR-amplified products of the litchi *LcGA2ox6* gene were employed as target fragments and ligated with the plant expression vector pCAMBIA2300 via homologous recombination. This methodology successfully facilitated the construction of plant overexpression vectors for the *LcGA2ox6* genes. Subsequently, genetic transformation was conducted in *Arabidopsis thaliana* (Columbia ecotype) utilizing the floral dip method mediated by *Agrobacterium tumefaciens*. T_0_ seeds underwent surface sterilization and were sown on Murashige and Skoog (MS) agar plates supplemented with 50 mg/L kanamycin for selection, resulting in the isolation of eight independent kanamycin-resistant lines.

**Figure 10:**
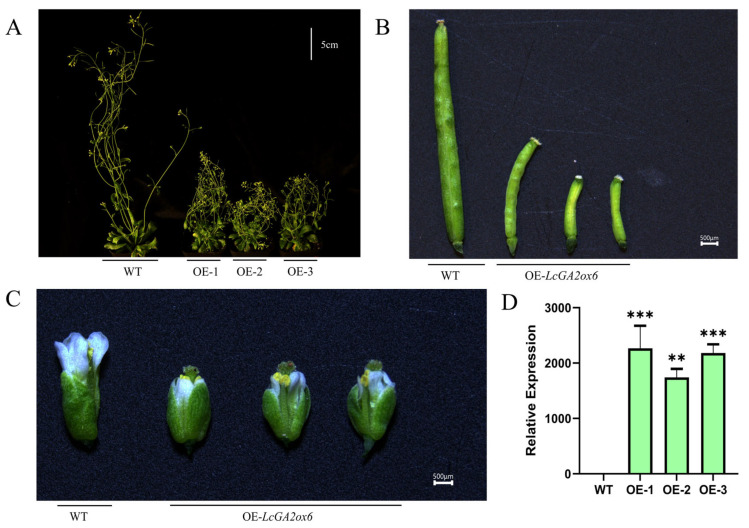
Phenotypic Observation and Expression Identification of *LcGA2ox6* Overexpressing Plants. (**A**) Phenotypic observation of wild-type and 52-day-old *LcGA2ox6*-transgenic *Arabidopsis thaliana*; (**B**) Silique phenotype of *Arabidopsis thaliana*; (**C**) Floret phenotype of *Arabidopsis thaliana*; (**D**) Expression level of *LcGA2ox6* in transgenic *Arabidopsis thaliana* detected by RT-qPCR (** *p* < 0.01, *** *p* < 0.001, based on One-way analysis of variance combined with Dunnett’s multiple comparison test).

Seeds from the T_0_ plants of these eight independent events were harvested separately to produce the T_1_ generation. Continued resistance screening was conducted to eliminate false-positive plants, and five lines exhibiting stable heritable resistance markers were retained. RT-qPCR analysis was performed on the T_1_ plants of these five lines, and those with a relative expression level of the *LcGA2ox6* gene exceeding five times that of the wild type were selected, yielding three high-expression candidate lines. Through an examination of the phenotypic consistency in the T_2_ generation of three candidate lines, we successfully identified three homozygous lines exhibiting stable heredity, consistent phenotypes, and high expression levels of the target gene, which were subsequently utilized for further functional verification experiments (Figure 10D, Figure S3 and Table S16).

Phenotypic observations revealed that, after 52 days of growth under standard conditions, the transgenic plants exhibited significant dwarfism compared to the wild type (WT) (Figure 10A and Table S10). Additionally, the length of the small flowers was markedly reduced (Figure 10C and Table S11), and the attachment position of stamens (anthers) was significantly lower than that of pistils (stigmas) (Figure S2 and Table S12a,b,c), impeding the effective transfer of pollen to stigmas. This was accompanied by self-sterility, as evidenced by the failure of siliques to develop normally following self-pollination under natural conditions, resulting in short, shrunken, and sterile characteristics (Figure 10B and Tables S13a,b and S14).

These findings suggest that the overexpression of *LcGA2ox6* can inhibit both vegetative and reproductive development in *Arabidopsis thaliana*, thereby disrupting the normal development of floral organs and fruits.

## 3. Discussion

In litchi production, the instability of flowering and the unbalanced male-to-female flower ratio are significant constraints on yield improvement, rendering the investigation of their sex differentiation mechanisms both theoretically and practically significant [1,30]. Accumulating evidence indicates that GAs serve as central regulators of floral sex determination, orchestrating developmental switches through the modulation of GA signaling components such as the *GA2ox* family. As a key enzyme in GA catabolism, GA2ox primarily functions to regulate GA homeostasis by degrading active GAs, thereby influencing plant growth and development and participating in GA feedback regulation to maintain physiological dynamic balance [2]. Nevertheless, the properties and biological roles of litchi *GA2ox* genes, especially regarding floral development, are still not well understood. Compared to herbaceous model plants such as *Arabidopsis* and rice, woody fruit trees like litchi exhibit longer growth cycles, more complex floral organ development mechanisms, and unique sex differentiation regulatory networks. Therefore, systematic identification and functional validation of the *LcGA2ox* family are essential, as their functions cannot be reliably inferred from model species.

This study identified nine *GA2ox* genes in litchi, all of which contain the conserved 2OG-Fe (II)_Oxy and DIOX_N domains (Figure 3C), thereby providing a robust foundation for the identification of the *GA2ox* gene family. The number of *GA2ox* genes in litchi is comparable to that in *Arabidopsis* (seven genes) and rice (nine genes). Phylogenetic analysis revealed that *LcGA2ox4/6/7* closely cluster with members of the *Arabidopsis* C_19_-GA2ox-I subgroup, specifically *AtGA2ox4/6* [31,32], suggesting conserved roles in the metabolism of GA inactivation. These genes primarily target active GAs or their direct precursors (e.g., GA_1_, GA_4_) to precisely regulate the levels of active GA through catalyzed inactivation [32]. Meanwhile, *LcGA2ox1/2/3/5/9* cluster with members of the *Arabidopsis* and rice C_19_-GA2ox-II subgroup, including *OsGA2ox3/6* and *AtGA2ox1/2* [32,33,34], and share the same substrate type as C_19_-GA2ox-I, albeit with broader expression across multiple tissues. Notably, rice *OsGA2ox6* is known to regulate plant height [35], suggesting analogous functions for the corresponding litchi genes within this clade. *LcGA2ox8* forms a distinct branch with members of the *Arabidopsis* and rice C_20_-GA2ox-I subgroup [35,36,37,38], targeting primarily early GA synthesis precursors (e.g., GA12, GA53) to inhibit the conversion of precursors to active GAs, thereby regulating overall GA synthesis from the source [9].

Consistent with findings in other plant species [13,39,40], comparative analyses of gene structure and motif architecture revealed that phylogenetically closely related members share highly similar exon-intron organizations and conserved motif compositions, suggesting evolutionary conservation in structural organization (Figure 3B,D). Among the ten conserved motifs identified in all GA2ox proteins, motifs 1, 3, 7, and 10 are part of the 2OG-Fe(II)_Oxy domain, which is essential for GA2ox activity. This domain specifically catalyzes the 2β-hydroxylation of GA substrates through Fe^4+^=O intermediates, a crucial step in GA inactivation [41]. For instance, the grape enzyme *VvGA2ox7* degrades both C19 and C_20_-type GAs via this domain, functioning as a transitional enzyme [16]. While not directly catalytic, the N-terminal DIOX_N domain plays a critical role in supporting the 2OG-Fe(II)_Oxy domain through the stabilization of protein conformation and the enhancement of substrate binding affinity and specificity [10,40,42]. It is noteworthy that individual variations in the conserved motifs of the DIOX_N domain among family members may be associated with substrate recognition or the functional specificity of specific signal responses.

Gene tandem duplication represents a prevalent mechanism for the expansion of plant gene families. Duplicated genes tend to accumulate mutations while maintaining essential functions, such as the conservation of the DIOX_N domain, and may acquire novel functions. This process provides genetic resources that enable litchi to adapt to various growth and developmental requirements, including plant architecture regulation and fruit development [43]. Analysis of gene duplication has identified tandem duplication events, such as those involving *LcGA2ox2* and *LcGA2ox9*, as significant contributors to the expansion of the *LcGA2ox* gene family (Figure 4A). Comparative genomic studies have demonstrated that *LcGA2ox2/4/6/7/9* exhibit collinear relationships with a range of plant species, including those within the closely related Sapindaceae family, suggesting ancient evolutionary origins and the conservation of core functions. Nonetheless, variations in collinear extents suggest functional differentiation: genes with broad collinearity, such as *LcGA2ox9*, may fulfill fundamental GA metabolic functions, whereas those with narrow collinearity, such as *LcGA2ox6*, may have evolved specialized regulatory roles specific to litchi developmental processes, such as floral organ formation.

The analysis of promoter *cis*-elements revealed that *LcGA2ox* genes are abundant in regulatory elements, encompassing those responsive to light (e.g., Box 4), hormones (e.g., ABRE, CGTCA motif), and developmental processes (e.g., CAT-box) as illustrated in Figure 5. This indicates that their expression is meticulously regulated by light signals, diverse hormones such as ABA and MeJA, and meristem development programs, thereby contributing to the initiation and development of floral organs.

Using *Arabidopsis* homologs as references, predictions from the STRING database elucidated the potential functions and regulatory roles of LcGA2ox in GA metabolism and signaling pathways (Figure 6). LcGA2ox4 interacts with the GA receptor GID1B, promoting GA-dependent DELLA degradation and thereby regulating reproductive development and stem elongation [24]. LcGA2ox5 associates with the DELLA protein RGL2 and the stress response factor DREB1E/F, suggesting a potential integration of GA signaling and stress responses to regulate flower and fruit development as well as stress resistance [25,44]. LcGA2ox1/2/3/9 interact with the key GA synthesis enzyme KAO2, which may influence GA synthesis homeostasis through feedback mechanisms [26]. The interaction between LcGA2ox6/7 and the DELLA protein RGA may modulate GA signaling by stabilizing RGA, which acts as a transcriptional repressor of floral development genes in *Arabidopsis*; the subsequent degradation of RGA is a prerequisite for the initiation of normal stamen development [27]. LcGA2ox8 interacts with multiple upstream synthases and signaling components, and as a member of the C_20_-GA2ox-I subgroup, it is hypothesized to regulate early GA synthesis steps during developmental transitions or in response to stress [28,29].

Utilizing RNA-seq data from the ‘Feizixiao’ litchi across various tissues and developmental stages, it has been observed that the *LcGA2ox* gene family demonstrates distinct spatiotemporal expression specificity. This specificity likely contributes to tissue development by modulating local GA levels. Transcriptomic analysis of expression patterns in the pedicel, aril, seed, and pericarp revealed that *LcGA2ox4* and *LcGA2ox8* are highly expressed in these tissues, thereby regulating GA levels during litchi fruit development. These findings are consistent with studies in tomatoes, where the overexpression of the fruit-specific gene *SlGA2ox1* leads to reduced endogenous GA levels, subsequently affecting fruit weight, seed number, and germination rate [45]. *LcGA2ox8* plays a crucial role in regulating GA metabolism throughout the development of leaf buds, significantly contributing to morphogenesis and growth differentiation. *LcGA2ox4/5/9* exhibit coordinated activity during critical phases of leaf bud development, suggesting a synergistic role in developmental regulation. This mechanism is consistent with observations in other species, where GA2ox-mediated inactivation of active GA dampens signaling intensity in leaf buds [46,47,48]. In litchi, the expression of *LcGA2ox4* in the pericarp increases progressively across the three stages of pericarp coloration: green, yellow, and red. This suggests that *LcGA2ox4* may be involved in the physiological processes associated with pericarp ripening and coloration through its continuous regulation of GA metabolism. *LcGA2ox8* is active during the late stages of pericarp ripening, while *LcGA2ox5* specifically modulates GA levels during the green fruit stage. This is analogous to the transient overexpression of *PbGA2ox8* in pear, which reduces pericarp GA4 levels and induces anthocyanin accumulation [49].

Research indicates that in litchi male flowers, endogenous GA content increases prior to meiosis, reaches its peak during meiosis, and subsequently decreases during pollen development and maturation. Notably, fertile stamens exhibit lower GA levels compared to abortive pistils. Conversely, in female flowers, abortive stamens demonstrate a continuous decline in GA content throughout development, whereas fertile pistils exhibit an increase in GA levels [50]. During the late stages (M3 and M4) of male flower development, *LcGA2ox6* and *LcGA2ox7* exhibit specific high expression, functioning in the regulation of GA metabolism to ensure proper anther maturation and pollen grain formation. These genes also contribute to the establishment of distinct GA environments and energy allocation patterns between male and female flowers through their high expression in male and bisexual flowers and low expression in female flowers, thereby influencing the direction of litchi flower sex differentiation. Notably, all *LcGA2ox* genes exhibit low expression in female flower pistils, likely reflecting the requirement for elevated GA levels to sustain prolonged reproductive processes.

The construction of co-expression networks for litchi male and female flower development using Weighted Gene Co-expression Network Analysis (WGCNA) has elucidated the regulatory associations involving *LcGA2ox6/7*. A strong positive association was observed between the blue module and the advanced stages of male flower development(M3, M4), and the genes within this module are enriched in pathways related to plant hormone signal transduction and diterpenoid biosynthesis, specifically the GA synthesis precursor pathway. These findings indicate that *LcGA2ox6/7* are pivotal genes in the maturation of male flowers, functioning through gene networks that co-express with 29 transcription factors, each with a weight greater than 0.35. Notably, *LcGA2ox6/7* show a strong positive correlation with the floral organ identity class E gene *SEP1* and a strong negative correlation with *AGL1* and *SPT*, which are highly expressed in litchi female flowers [51]. *AGL1* is recognized as a marker gene for carpel development in *Arabidopsis* [52], while *SPT* is identified as a key regulatory factor in pistil morphogenesis [53]. These findings not only underscore the involvement of *LcGA2ox6/7* in male flower maturation but also offer new insights into the molecular regulation of litchi flower sex differentiation. Furthermore, they suggest that *AGL1* and *SPT* may be key candidate genes involved in female flower development. It is widely believed that LcGA2ox6/7 mediates the 2β-hydroxylation inactivation of bioactive GAs like GA_1_ and GA_4_, reducing GA levels in litchi male flowers during late development. This creates a male flower-specific environment with lower GA concentrations, which prevents the degradation of the DELLA protein RGA, leading to its accumulation. RGA accumulation directly binds to the promoters of AGL1 and SPT, repressing their transcription. AGL1, a carpel development marker, and SPT, a key regulator of pistil morphogenesis, show decreased expression, disrupting pistil development pathways and hindering normal pistil differentiation and maturation. Furthermore, RGA may boost SEP1 expression by relieving transcriptional repression or enhancing its regulatory mechanisms. Functioning as a class E floral identity gene, *SEP1* plays a pivotal role in organ identity determination through complex formation with class B and C genes. High expression of *SEP1* during late male flower development facilitates synergistic interactions with stamen development-related genes, such as those associated with pollen formation and anther dehiscence. This interaction reinforces the stamen-specific developmental program and facilitates the seamless progression of crucial processes, including pollen maturation and anther dehiscence. 

The expression pattern and co-expression network analyses performed in this study indicate that *LcGA2ox6* and *LcGA2ox7* demonstrate highly coordinated expression characteristics. These genes are specifically and predominantly expressed during the late developmental stage of litchi male flowers, and together they establish a co-expression network with transcription factors involved in floral sex differentiation. This observation implies that *LcGA2ox6* and *LcGA2ox7* may have functionally redundant or synergistic roles in regulating male flower maturation and sex differentiation. The full-length coding sequences (CDS) of *LcGA2ox6* and *LcGA2ox7* are both 1023 base pairs long and exhibit 99.3% sequence similarity, differing by only seven base pairs in the central region of the coding sequence, while the remaining sequences are completely identical. This high degree of homology presented challenges in designing primers capable of distinguishing between the two genes in the conserved regions at both ends. As a result, the final primer pairs were only able to efficiently amplify the *LcGA2ox6* gene, and were unsuccessful in specifically cloning the complete CDS of *LcGA2ox7* from litchi samples. To thoroughly investigate the biological functions of *LcGA2ox6*, this study successfully introduced the *LcGA2ox6* gene into wild-type *Arabidopsis thaliana* through genetic transformation. Through rigorous screening, three transgenic lines with elevated expression levels were identified. Phenotypic analyses demonstrated that, compared to wild-type plants, these transgenic lines exhibited a distinct dwarf phenotype (Figure 10A and Table S10), significantly reduced floret length (Figure 10C and Table S11), and a notably lower attachment position of stamens (anthers) relative to pistils (stigmas), which impeded effective pollen transfer to stigmas (Figure S2 and Table S12a,b,c). This was associated with self-sterility, as self-pollination under natural conditions led to siliques that failed to develop normally, characterized by short, shrunken, and sterile features (Figure 10B and Tables S13a,b and S14). These findings provide direct evidence for the regulatory role of *LcGA2ox6* in floral organ and fruit development, supporting previous reports on the gibberellin-mediated control of pistil formation, filament growth, and anther dehiscence [1].

Through a combination of genome-wide identification and multi-dimensional analysis, this research systematically elucidates the core characteristics and expression dynamics of the litchi *LcGA2ox* gene family. The findings reveal that while *LcGA2ox* members are highly conserved throughout evolution, they exhibit significant differentiation in terms of expression and function. Notably, the specific expression of *LcGA2ox6/7* during the late stage of male flower development, coupled with their negative correlation with key female flower genes *AGL1* and *SPT*, strongly indicates the central regulatory role of these genes in litchi sex differentiation. These results not only provide essential genetic resources for the detailed analysis of the litchi GA metabolic network but also establish a theoretical foundation for enhancing litchi flowering and reproductive traits through molecular approaches.

## 4. Materials and Methods

### 4.1. Identification of Members of the LcGA2ox Gene Family

The genetic resources for Litchi, encompassing DNA sequences, GFF files, coding sequences (CDS), and protein sequences, were acquired from the SapBase repository (available at http://www.sapindaceae.com/Download.html, last accessed on 10 September 2025). In parallel, *Arabidopsis* genomic data was downloaded from The *Arabidopsis* Information Resource (TAIR) platform (https://www.arabidopsis.org/, accessed on 10 September 2025). Reference protein sequences of *GA2ox* family members from *Arabidopsis* and rice were extracted from the Plant Transcription Factor Database (PlantTFDB, https://planttfdb.gao-lab.org/, accessed on 10 September 2025) and employed as search templates to identify similar sequences in the litchi proteome through BLAST analysis implemented in TBtools-II (v2.210). Additionally, the 2OG-Fe(II)_Oxy domain profile (PF03171) was obtained from InterPro (https://www.ebi.ac.uk/interpro/entry/pfam/PF03171/, accessed on 11 September 2025), followed by homology detection using HMMER (v3.4) software to screen for putative *GA2ox* family genes in litchi.

The findings from BLAST and HMMER investigations were integrated to identify conserved protein regions. Through the NCBI’s Conserved Domain Database (CDD) online platform (https://www.ncbi.nlm.nih.gov/Structure/cdd/wrpsb.cgi/, accessed on 11 September 2025), sequences missing the characteristic 2OG-Fe(II)_Oxy domain were eliminated, thus confirming the complete set of litchi *GA2ox* gene family members. Various physicochemical properties including molecular mass, amino acid count, theoretical pI, and protein stability were calculated using ExPASy’s ProtParam tool (https://web.expasy.org/protparam/, accessed on 11 September 2025). Cellular compartmentalization predictions were performed via the WoLF PSORT prediction server (https://wolfpsort.hgc.jp/, accessed on 11 September 2025).

### 4.2. Construction of the Phylogenetic Tree of LcGA2ox Genes

The phylogenetic analysis was conducted using MEGA11 (v11.0.9) software to examine the evolutionary relationships among *LcGA2ox* gene family members in litchi, along with GA2ox homologs from *Arabidopsis* and rice. Protein sequence alignment was performed through ClustalW, followed by phylogenetic tree construction employing the Neighbor-joining algorithm. To ensure robustness, 1000 bootstrap replications were implemented while maintaining all other parameters at their standard configurations. The resulting phylogenetic relationships were then visualized.

### 4.3. Analysis of Conserved Domains, Gene Structure, and Conserved Motifs of LcGA2ox Genes

The amino acid sequences of litchi *GA2ox* genes were analyzed using the NCBI’s Web CD-search Tool (https://www.ncbi.nlm.nih.gov/Structure/cdd/wrpsb.cgi, accessed on 12 September 2025) to identify conserved domains, with the findings being graphically represented through TBtools-II’ Gene Structure View function. Additionally, the genomic organization of *LcGA2ox* genes was examined and illustrated using the same visualization tool in TBtools-II, incorporating data from the litchi genome annotation file. For motif analysis, the MEME online platform (https://meme-suite.org/meme/, accessed on 12 September 2025) was employed to identify ten conserved protein motifs, which were subsequently displayed using TBtools’ visualization capabilities.

### 4.4. Chromosomal Distribution and Synteny Analysis of LcGA2ox Genes

The study utilized genomic sequences and annotation data from litchi along with five other Sapindaceae species (*Dimocarpus longan*, *Nephelium lappaceum*, *Sapindus mukorossi* Gaertn., *Xanthoceras sorbifolium*, and *Acer pseudosieboldianum*), as well as *Arabidopsis* and rice, to conduct comparative genomic analyses. These datasets enabled the examination of both intra-specific genomic synteny within litchi and inter-specific syntenic relationships between litchi and the other selected species. The investigation employed the One Step MCScanX module in TBtools-II software to analyze the evolutionary patterns of *GA2ox* genes. Genomic information for the five Sapindaceae plants was acquired from the SapBase repository (available at http://www.sapindaceae.com/Download.html, accessed on 25 September 2025), whereas the *Arabidopsis* and rice genomic data were sourced from the Ensemble plant database (accessible via http://plants.ensembl.org/index.html/, accessed on 20 September 2025).

### 4.5. Analysis of Cis-Acting Elements in LcGA2ox Gene Promoters and Prediction Analysis of Protein–Protein Interactions

Using the Gtf/Gff3 sequence extraction feature within TBtools-II software, researchers isolated a 2000 nucleotide segment located upstream from the initiation codon for each *LcGA2ox* gene, identifying this region as the potential promoter sequence. These promoter sequences were subsequently analyzed through the Plant CARE online resource (http://bioinformatics.psb.ugent.be/webtools/plantcare/html/, accessed on 14 September 2025) to identify potential regulatory elements, with the findings graphically represented using TBtools’ Simple BioSequence Viewer functionality. For protein interaction studies, the STRING database (https://cn.string-db.org/, accessed on 28 September 2025) was utilized, while supplementary protein data was retrieved from the UniProt knowledgebase (https://www.uniprot.org/, accessed on 28 September 2025).

### 4.6. Analysis of Expression Pattern in LcGA2ox Genes in Litchi

The transcriptomic datasets employed in this research comprised three publicly available datasets and one novel unpublished dataset, including: Transcriptome profiles of various ‘Feizixiao’ litchi tissues (callus, root, leaf, flower, fruit, bud, pedicel base, aril, seed, and pericarp) collected from natural growth conditions, each with triplicate biological samples (NCBI accession: PRJNA747875; available at https://www.ncbi.nlm.nih.gov/bioproject/PRJNA747875/, accessed on 1 December 2025) [54]; Gene expression data from different developmental stages of ‘Feizixiao’ litchi (pedicel, pulp, seed, pericarp, and young fruit) under natural growth conditions, with three biological replicates for each tissue type (NCBI accession: PRJNA299841; available at https://www.ncbi.nlm.nih.gov/bioproject/PRJNA299841/, accessed on 1 December 2025) [55]; RNA sequencing datasets were obtained from ‘Feizixiao’ litchi pericarp tissues exhibiting green, yellow, and red coloration, with each color group containing three biological replicates (NCBI BioProject: PRJNA261000; available at https://www.ncbi.nlm.nih.gov/bioproject/PRJNA261000/, accessed on 1 December 2025) [56]; Transcriptomic profiles from various developmental phases of both male and female flowers in ‘Feizixiao’ litchi plants grown under natural conditions, with triplicate samples for each stage; Gene expression data collected from distinct floral structures of ‘Feizixiao’ litchi, comprising three replicate samples per organ type (BioProject accession: PRJNA756275; accessible via https://www.ncbi.nlm.nih.gov/bioproject/PRJNA756275/, accessed on 1 December 2025) [56].

### 4.7. Construction and Analysis of Weighted Gene Co-Expression Networks (WGCNA)

To better understand the relationships and control mechanisms of *LcGA2ox* genes, researchers utilized WGCNA shiny to examine RNA sequencing data from male and female litchi flowers at various growth phases. The investigation involved grouping genes into specific co-expression clusters through dynamic tree segmentation techniques. By evaluating how these genetic clusters related to sample characteristics and observing their expression trends in diverse samples, scientists were able to pinpoint crucial genes. For network visualization and diagram creation, the team implemented Cytoscape version 3.10.1 software.

### 4.8. Transformation and Screening of LcGA2ox Transgenic Plants

The *LcGA2ox* gene sequence was cloned into the pCAMBIA2300-GFP plasmid following digestion with BamHI and SalI endonucleases, resulting in the pCAMBIA2300-GFP-*LcGA2ox* expression construct. Upon successful sequence confirmation, the engineered plasmid was mobilized into the *Agrobacterium tumefaciens* GV3101 host strain. *Arabidopsis* plants (Columbia background) were grown under controlled conditions with 21 ± 1 °C day/night temperatures and 16 h photoperiods. Genetic transformation was performed using the floral dip technique [57], employing wild-type plants as reference controls. T_0_ seeds were collected and selected on kanamycin-containing MS medium (50 mg/L), with transgenic plants subsequently confirmed through PCR analysis. Homozygous T_3_ lines were chosen for further characterization and functional studies. Both transgenic and control *Arabidopsis* plants at 52 days post-germination were harvested for subsequent experimental procedures.

The quantification of *LcGA2ox6* transcript abundance was performed through quantitative real-time polymerase chain reaction (qRT-PCR) analysis. The specific oligonucleotide sequences employed for plasmid assembly are provided in Supplementary Table S15.

### 4.9. RNA Extraction and RT-qPCR Analysis

The floral tissues of transgenic *Arabidopsis* plants overexpressing *LcGA2ox6* were subjected to RNA isolation employing the Polysaccharide and Polyphenol Plant RNA Extraction Kit (Huayueyang, Beijing, China), following the standard protocols provided by the manufacturer. RNA purity and quantity were assessed with a NanoDrop™ One/OneC spectrophotometer (Thermo Fisher Scientific, Waltham, MA, USA), followed by cDNA synthesis utilizing the Revert Aid First Strand cDNA Synthesis Kit (Thermo Fisher Scientific, USA).

Semi-quantitative PCR (semi-PCR) was performed to verify the expression of *LcGA2ox6* in transgenic *Arabidopsis* lines. The reaction system was prepared with KOD One™ PCR Master Mix (Toyobo Co., Ltd., Osaka, Japan) from Toyobo, specific primers for *LcGA2ox6* and the internal control gene *AtActin*, cDNA template, and RNase-free water. The thermal cycling parameters were set as follows: initial denaturation at 94 °C for 1 min; followed by 28 amplification cycles, each consisting of 98 °C denaturation for 10 s, 58 °C annealing for 5 s, and 68 °C extension for 15 s; and a final extension at 68 °C for 5 min. PCR products were separated by 1% agarose gel electrophoresis, stained with nucleic acid dye, and visualized using a gel imaging system; the band intensity was used to reflect the relative expression level of the target gene.

Quantitative real-time PCR analysis was conducted on a LightCycler 480 II system (Roche, Basel, Switzerland) with ChamQ Universal SYBR qPCR Master Mix (Vazyme, Nanjing, China). The internal control for normalization was *AtActin* from *Arabidopsis*, with the thermal cycling parameters consisting of an initial denaturation step at 95 °C for 120 s, followed by 40 amplification cycles (95 °C for 10 s, 58 °C for 30 s), and concluding with a melting curve analysis (95 °C for 15 s, 60 °C for 60 s, 95 °C for 15 s). Three independent biological samples and three technical replicates were analyzed for each experimental condition to determine the comparative expression levels of target genes. The relative expression levels were determined through the 2^−ΔΔCt^ calculation approach [58].

The specific primer sequences employed in the semi-PCR and RT-qPCR experiments can be found in Supplementary Table S15.

## 5. Conclusions

Nine *LcGA2ox* family members were successfully identified in litchi, each containing the characteristic 2OG-Fe(II)_Oxy and DIOX_N domains typical of the *GA2ox* family. Through bioinformatics analysis, the physicochemical properties, gene structures, and evolutionary characteristics of these genes were elucidated. Protein–protein interaction predictions suggest that *LcGA2ox8* may serve as a key regulatory component in GA metabolism and signaling pathways. Analysis of expression patterns revealed distinct tissue-specific and spatiotemporal expression, highlighting functional differentiation during litchi organ development. Notably, *LcGA2ox6* and *LcGA2ox7* are predominantly expressed during male flower development and exhibit a strong negative correlation with the core female flower genes *AGL1* and *SPT*, indicating their role as key regulators of male flower maturation and sex differentiation in litchi. The inhibitory effect of *LcGA2ox6* on plant growth and development was further validated by overexpression assays in *Arabidopsis*. These findings lay a significant theoretical foundation and provide essential genetic resources for a comprehensive understanding of the GA metabolic network in litchi. This study seeks to uncover key regulators of sex differentiation and enhance flowering and reproductive performance in litchi using molecular breeding strategies.

## Figures and Tables

**Table 1:** Summary information of the *LcGA2ox* family.

Gene Name	Gene ID	Number of Amino Acid	Molecular Weight (kDa)	Theoretical pI	Instability Index	Aliphatic Index	Grand Average of Hydropathicity	Sub-Cellular Location
*LcGA2ox1*	*LITCHI006115*	348	39.81	6.56	40.7	82.64	−0.311	cytosol
*LcGA2ox2*	*LITCHI013387*	347	39.96	6.22	51.29	73.6	−0.453	nucleus
*LcGA2ox3*	*LITCHI013389*	347	39.96	6.22	51.29	73.6	−0.453	nucleus
*LcGA2ox4*	*LITCHI014295*	371	41.01	6.07	50.94	84.07	−0.053	chloroplast
*LcGA2ox5*	*LITCHI025158*	347	39.70	8.62	38.99	82.28	−0.499	cytosol
*LcGA2ox6*	*LITCHI026603*	340	38.00	8.87	39.98	86.88	−0.303	chloroplast
*LcGA2ox7*	*LITCHI026618*	340	37.96	9	42.41	87.18	−0.295	chloroplast
*LcGA2ox8*	*LITCHI028803*	332	37.41	8.4	35.21	86.63	−0.272	cytosol
*LcGA2ox9*	*LITCHI029529*	355	40.63	6.23	43.38	78.23	−0.354	cytosol

## Data Availability

The original contributions presented in the study are included in the article and Supplementary Materials; further inquiries can be directed to the corresponding authors.

## Supplementary Materials

The following supporting information can be downloaded at: https://www.mdpi.com/article/10.3390/plants15060914/s1, Table S1: Expression patterns of LcGA2ox genes in different litchi tissues; Table S2: Expression patterns of *LcGA2ox* genes in different litchi fruit organs; Table S3: Expression patterns of *LcGA2ox* genes in four developmental stages of leaf buds; Table S4: Expression patterns of *LcGA2ox* genes during litchi pericarp coloring; Table S5: Expression patterns of *LcGA2ox* genes during the development of litchi male and female flowers; Table S6: Expression patterns of *LcGA2ox* genes in different litchi floral organs; Table S7: The specific values of co-expression weights between 29 transcription factors and the *LcGA2ox6/7* genes; Table S8: Co-expressed genes of *LcGA2ox6/7*; Table S9: Expression patterns of *LcGA2ox6/7* and their co-expressed genes; Table S10: Plant height statistics of WT and OE-*LcGA2ox6*; Table S11: Flower Length Group statistics of WT and OE-*LcGA2ox6*; Table S12a: Stigma Height Group statistics of WT and OE-*LcGA2ox6*; Table S12b: Anther Height Group statistics of WT and OE-*LcGA2ox6*; Table S12c: Stigma-anther distance Group statistics of WT and OE-*LcGA2ox6*; Table S13a: Silique Number Group statistics of WT and OE-*LcGA2ox6*; Table S13b: Aborted Silique Number Group statistics of WT and OE-*LcGA2ox6*; Table S14: Silique Length Group statistics of WT and OE-*LcGA2ox6*; Table S15: List of the primers used in this study; Table S16: qRT-PCR validation of *LcGA2ox6*-overexpressing *Arabidopsis thaliana*; Figure S1: GO and KEGG enrichment analyses of co-expressed genes in the blue module; Figure S2: Stamens and pistils of florets in *LcGA2ox6*-overexpressing *Arabidopsis thaliana*; Figure S3: Semi-quantitative PCR analysis of *LcGA2ox6*-overexpressing *Arabidopsis thaliana* lines.
