# Supplementary material for: Genome-Wide Identification of the LcGA2ox Gene Family in Litchi (Litchi chinensis Sonn.) and Its Functional Analysis in Gibberellin Metabolism and Reproductive Development
Source: Plants (Basel). 2026 Mar 16;15(6):914. doi: 10.3390/plants15060914 (PMC13030733; doi:10.3390/plants15060914)
Supplement: Supplementary file 1 [file plants-15-00914-s001.zip › Supplementary Figure.pdf]

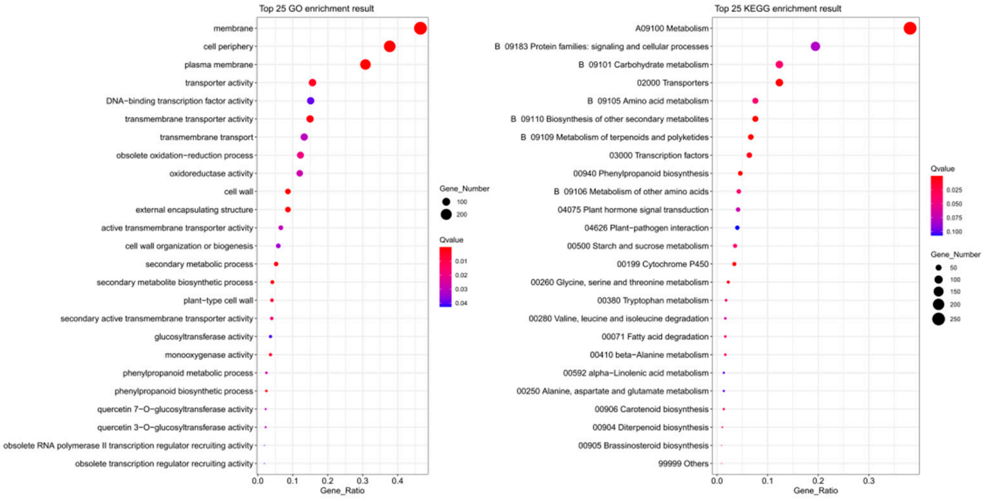

Figure S1. GO and KEGG enrichment analyses of co-expressed genes in the blue module

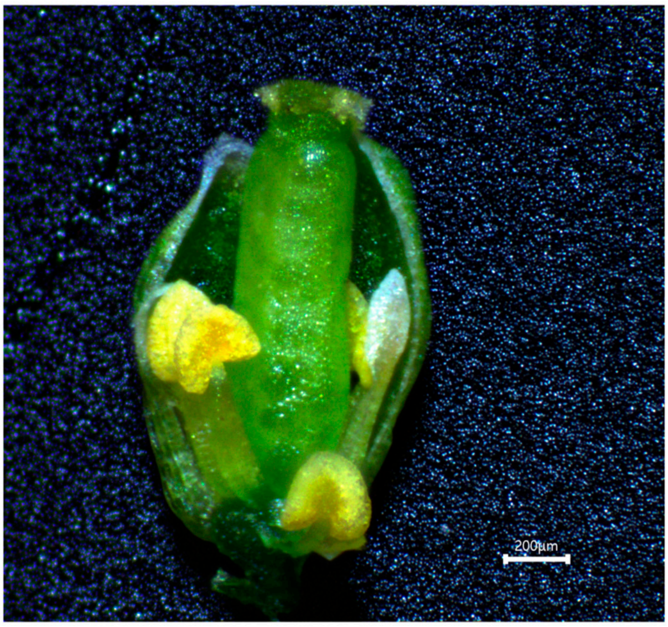

Figure S2. Stamens and pistils of florets in *LcGA2ox6*-overexpressing *Arabidopsis thaliana*

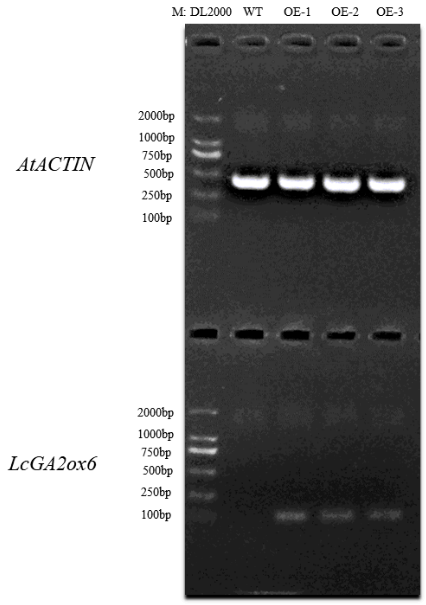

Figure S3. Semi-quantitative PCR analysis of *LcGA2ox6*-overexpressing *Arabidopsis thaliana* lines
